# National

**Published:** 1897-03

**Authors:** 


*National.* In a recent order drawing the cattle quarantine-line, to take effect on February 15th and be operative until November 15th, the Secretary of Agriculture announces that a contagious and infectious disease, known as splenetic or Southern fever, exists among cattle in California, Indian Territory, Oklahoma, Texas, Arkansas, Louisiana, and all the Southern States east of the Mississippi River and south of Kentucky, West Virginia, and Maryland. This quarantine-line has since been modified in Texas, Oklahoma, Arkansas, Virginia, and North Carolina to conform to quarantine-lines established by those States; and should other States now under the ban adopt a new line and regulations by legislative enactment to the satisfaction of the department, special orders will be issued accordingly. During the period specified in the regulations no cattle can be transported from the area south of the Federal quarantine-line to the north, east, or west of that line except by rail for immediate slaughter. Wherever cattle from the quarantined district are fed or watered in transit, no other cattle must be admitted, and the cars that carried the infected cattle and pens where they are temporarily detained must be cleansed and disinfected. Under the regulations Mexican cattle are admitted to remain below the Federal quarantine-line, and can be transported across it for immediate slaughter or upon a special permit from the inspectors of the Bureau of Animal Industry. The Agricultural Department has also issued notice that cattle infested with the Southern cattle-tick disseminate the Texas fever, and will, therefore, be considered as infectious cattle and be subject to the rules and regulations governing the movement of Southern cattle.—*Breeders' Gazette*, February 10, 1897.

